# The effect of hyperglycemic peak induced by oral glucose tolerance test on the oxidant and antioxidant levels

**DOI:** 10.3906/sag-1905-106

**Published:** 2019-12-16

**Authors:** Tekin YILDIRIM, Yeşim GÖÇMEN, Zeynep Tuğba OZAN, Elif BÖREKÇİ, Elif TURAN, Yalçın ARAL

**Affiliations:** 1 Department of Internal Medical Sciences, Faculty of Medicine, Yozgat Bozok University, Yozgat Turkey; 2 Department of Biochemistry Sciences, Faculty of Medicine, Yozgat Bozok University, Yozgat Turkey; 3 Department of Internal Medical Sciences, Faculty of Medicine, Celal Bayar University, Manisa Turkey; 4 Department of Endocrinology Sciences, Faculty of Medicine, Yozgat Bozok University, Yozgat Turkey

**Keywords:** Oral glucose tolerance test, oxidative parameters, antioxidative parameters, oxidative stress

## Abstract

**Background/aim:**

The possibility of adverse effects of the oral glucose tolerance test (OGTT) carried out for the screening of gestational diabetes among pregnant women and fetuses is a frequently discussed topic. The purpose of this study was to investigate the effects of the hyperglycemia peak during OGTT on the levels of oxidants and antioxidants in the body.

**Materials and methods:**

Eighty individuals who applied to the Outpatient Clinic with suspected diabetes and OGTT indication were included in the study. Glucose, total oxidant capacity status (TOS), total antioxidant capacity (TAS), superoxide dismutase (SOD), and lipid hydroperoxide (LOOH) levels were tested on blood samples collected from these individuals at 0, 60, and 120 min during the OGTT carried out with 75 g of glucose. Oxidative stress index (OSI) was calculated as the ratio of TOS to TAS.

**Results:**

While the oxidative parameters TOS and LOOH were significantly increased at 60. min of OGTT, only LOOH was significantly increased at 120. min of OGTT. Significant decreases in antioxidative parameters (TAS, SOD) were observed at 60. and 120. min of the OGTT and OSI was significantly increased at 60. and 120. min of the OGTT.

**Conclusion:**

Oxidative stress parameters were increased and antioxidative parameters were decreased during the OGTT. However, more extended studies are required to determine the effects of the increased oxidative stress on pregnant women and fetuses.

## 1. Introduction

Oxidative stress has an important role in the pathogenesis and late complications of diabetes. As a result of having one or more unshared electron, free radicals are very reactive and they tend to grab electrons from other atoms or molecules to fill their outer energy levels [1]. In diabetes mellitus, oxidative stress can occur because of the production of reactive oxygen species (ROS) including superoxide (O2‒) radical, hydroxide radical (HO‒), and hydrogen peroxide (H_2_O_2_) in high levels and/or inadequacy of antioxidant defense systems [2]. Increase in ROS production is related to protein glycosylation and/or autoxidation of glucose under hyperglycemic conditions. The reason for the inadequacy of neutralization of free radicals is related to the inadequacy of enzymatic and nonenzymatic radical scavengers (antioxidants) [3].

Glucose oxidation, nonenzymatic protein glycation, and the following oxidative degradation of glycosylated proteins in diabetes result in the formation of free radicals in excessive amounts. Free radicals formed in high amounts in general, simultaneously with the reduction in the efficiency of antioxidant defense mechanisms, damage the cellular organelles and enzymes, and cause lipid peroxidation and increase in insulin resistance. Hence, oxidative stress may lead to the occurrence of diabetic complications [4].

Furthermore, a high concentration of glucose in diabetes causes sorbitol production through the polyol pathway. Since nicotinamide adenine dinucleotide phosphate

(NADPH) is used for glucose reductase enzyme activity in this pathway, intracellular NADPH will be consumed in diabetes. NADPH is required to transform oxidized glutathione to a reduced form and for the synthesis of nitric oxide (NO). Therefore, the active sorbitol pathway and consequently the absence of NADPH will mean the limitation of the antioxidant capacity [4,5].

Diabetes mellitus is a prevalent disease group in the general population, and sometimes the oral glucose tolerance test (OGTT) is used for diagnostic purposes. The possibility of the adverse effects of the OGTT carried out for the screening of gestational diabetes among pregnant women and fetuses is a frequently discussed topic. The purpose of this study was to investigate the effects of the hyperglycemia peak during the OGTT on the levels of oxidants and antioxidants in the body and the balance between them in the body.

## 2. Materials and methods

This prospective, cross-sectional study was conducted between January 1, 2018, and August 29, 2018, at the outpatient clinics of the Internal Medicine and Endocrinology Departments in a tertiary hospital.

The study was approved by the Local Ethics Committee of the Faculty of Medical Sciences of Yozgat Bozok University (Protocol Number: 22.04.2016/42). We adhered to the principles of the Helsinki Declaration during the study and written informed consent was obtained from all participants.

### 2.1. Subjects

Eighty individuals who applied to the Internal Diseases and Endocrinology and Metabolism Diseases Outpatient Clinic with suspected diabetes and OGTT indication were included in the study. These individuals were between 18 and 65 years old and had no previous disease. Glucose, total oxidant capacity status (TOS), total antioxidant capacity (TAS), superoxide dismutase (SOD), and lipid hydroperoxide (LOOH) levels were tested on blood samples collected from these individuals at 0., 60. and 120. min during the OGTT carried out with 75 g of glucose. Oxidative stress index (OSI) was calculated as the ratio of TOS to TAS (OSI = TOS/TAS). Changes in these parameters obtained at 0., 60., and 120. min of the OGTT and the oxidant stress index (OSI) were statistically evaluated. 

In addition, patients were divided into 3 groups based on the OGTT results: Group 1: Normal (baseline glucose level < 110 mg/dL and glucose level at 120. min of the OGTT < 140 mg/dL); Group 2: Individuals with impaired fasting glucose and/or impaired glucose tolerance (glucose level between 100 mg/dL and 126 mg/dL at baseline and/or glucose level between 140 mg/dL and 200 mg/dL at 120. min); Group 3: Diabetic individuals (baseline glucose level ≥ 126 mg/dL or glucose level ≥ 200 mg/dL at 120. min of the OGTT). Comparison of these groups was performed in terms of oxidative and antioxidative parameters.

### 2.2. Blood collection and preparation

Venous blood samples were collected from an antecubital vein of each patient after a 12-h overnight fast, and a 10 mL sample of venous blood was placed into a biochemistry tube. Blood samples were withdrawn and sera were separated with centrifugation at 3000 rpm for 10 min. All material was stored at –80 °C until analysis.

### 2.3. Analysis of blood samples

Serum TOS, TAS, LOOH, and SOD were determined with a Rel Assay Diagnostics Kit (Mega Tıp, Gaziantep, Turkey) developed by Erel, and OSI values were calculated.

### 2.4. Determination of total antioxidant status (TAS)

TAS was measured in the serum by the generation of 2,2′-azino-di-(3-ethylbenzothiazoline sulfonate) (ATBS) radical cation using a commercial kit according to the manufacturer’s manual.

### 2.5. Determination of total oxidant status (TOS)

TOS was measured as described by the manufacturer’s protocol. In this method, the oxidants present in the sample oxidized the ferrous ion-o-dianisidine complex to ferric ion. Ferric ion produces a colored complex with xylenol orange in an acidic medium. The color intensity, which can be measured spectrophotometrically, is related to the total amount of oxidant molecules present in the sample. The assay was calibrated with hydrogen peroxide and the results were expressed in terms of μmol H_2_O_2_ equivalent/L of the serum.

### 2.6. Measurement of LOOH levels

Serum LOOH levels were measured with ferrous ion oxidation-xylenol orange (FOX-2) assay, which involves the oxidation of ferrous ion to ferric ion by various oxidants. The ferric ion is then measured with xylenol orange. The LOOH levels are reduced by the application of triphenylphosphine (TPP), which is a specific reductant for lipids. LOOH levels can be estimated as the difference in values in the absence or the presence of TPP. 

### 2.7. Calculation of oxidative stress index (OSI)

The TOS/TAS ratio was used as the oxidative stress index (OSI) and was calculated as follows: OSI (arbitrary units) = [(TOS, μmol H_2_O_2_/L) / (TAS, mmol Trolox equiv./L)].

### 2.8. Determination of superoxide dismutase (SOD) activity

The total SOD activity was determined in the serum using the SOD Activity Assay Kit according to the manufacturer’s instructions. SOD was measured by utilizing a tetrazolium salt for detection of superoxide radicals generated by xanthine oxidase and hypoxanthine. Measurements of TOS, TAS, LOOH, and SOD were performed on a Multiscan GO microplate reader (Thermo Scientific, USA).

### 2.9. Statistical analysis

All analyses were performed using SPSS version 23.0 (IBM Corp., Armonk, NY, USA). The suitability of the data for normal distribution was evaluated by Kolmogorov–Smirnov and Shapiro–Wilk tests. Quantitative data showing normal distribution are given as mean and standard deviation. In comparison between groups, one-way ANOVA was used, and repeated measures ANOVA was used for comparison of repeated measures. In the case of a difference, the post hoc Bonferroni test was used to find out which group(s) caused the difference. The relationships between the variables were evaluated by Pearson correlation test. Statistical significance level was taken as P <0.05.

## 3. Results

The OGTT was applied to 80 individuals with OGTT indication in this study. It was found that glucose, TOS, LOOH, and OSI reached the highest values at 60. min, and the values at 120. min were lower than those at 60. min while they remained high compared to the baseline. It was also found that TAS and SOD values were lowest at 60. min and increased at 120. min, while they remained lower as compared to the baseline (Table 1).

**Table 1:** TOS, LOOH, TAS, SOD, and OSI levels at the baseline, 60. and 120. min in OGTT.

	Time (min)			P*
	0 (n: 80)	60 (n: 80)	120 (n: 80)	
Glucose (mg/dL)	103.33 ± 19.02	188.70 ± 54.93	145.85 ± 50.67	<0.001
TOS	1.77 ± 0.22	2.64 ± 0.39	1.89 ± 0.23	<0.001
LOOH	16.40 ± 2.56	26.97 ± 3.66	19.16 ± 4.07	<0.001
TAS	2.20 ± 0.86	1.61 ± 0.43	1.85 ± 0.47	<0.001
SOD	1.81 ± 0.23	1.50 ± 0.18	1.68 ± 0.25	<0.001
OSI	0.89 ± 0.29	1.80 ± 0.64	1.09 ± 0.33	<0.001

* Repeated measures ANOVA. TOS (μmol H_2_O_2_/L): Total oxidant capacity status; LOOH (μmol/L): Lipid hydroperoxide; TAS (mmol Trolox equiv./L): Total antioxidant capacity; SOD (U/mL): Superoxide dismutase; OSI: Oxidative stress index; OGTT: Oral glucose tolerance test.

There was no statistically significant difference between the TOS levels at baseline and 120. min (P = 0.06), while there were statistically significant differences between the levels of all the other parameters at the studied time points (P < 0.05) (Table 2).

**Table 2:** Comparison of glucose, TOS, LOOH, TAS, SOD and OSI at the baseline, 60. and 120. min in OGTT.

	I (min)	J (min)	Mean difference (I-J)	P*
Glucose (mg/dL)	0	60	–85.38	<0.001
		120	–42.53	<0.001
	60	120	42.85	<0.001
TOS	0	60	–0.87	<0.001
		120	–0.13	0.060
	60	120	0.74	<0.001
LOOH	0	60	–10.57	<0.001
		120	–2.77	<0.001
	60	120	7.80	<0.001
TAS	0	60	0.60	<0.001
		120	0.35	<0.001
	60	120	–0.24	<0.001
SOD	0	60	0.31	<0.001
		120	0.14	<0.001
	60	120	–0.18	<0.001
OSI	0	60	–0.91	<0.001
		120	–0.21	<0.001
	60	120	0.70	<0.001

* Bonferroni post hoc test. TOS (μmol H_2_O_2_/L): Total oxidant capacity status; LOOH (μmol/L): Lipid hydroperoxide; TAS (mmol Trolox equiv./L): Total antioxidant capacity; SOD (U/mL): Superoxide dismutase; OSI: Oxidative stress index; OGTT: Oral glucose tolerance test.

All the parameters (glucose, TOS, LOOH, TAS, SOD, and OSI) were compared at baseline, 60. min, and 120. min of the OGTT in the 3 groups. A statistically significant difference was found between the glucose levels (P < 0.05), while no statistically significant differences were found between the other parameters (P > 0.05). 

When the relationships between glucose and TOS, LOOH, TAS, SOD, and OSI were evaluated, statistically significant positive correlations were found between glucose and TOS, LOOH, and OSI (P < 0.001, r = 0.572; P < 0.001, r = 0.501; P < 0.001, r = 0.470, respectively) and statistically significant negative correlations were found between glucose and TAS and SOD (P < 0.001, r = –0.243 and P = 0.012, r = –0.162, respectively).

However, we were unable to carry out intragroup evaluations because of the small number of patients in the groups (25 patients in group 2 and 13 patients in group 3).

## 4. Discussion

We aimed to evaluate the effect of the hyperglycemic peak during the OGTT on oxidative stress markers and we found that oxidative stress parameters were increased and antioxidative parameters were decreased during the OGTT in this study. 

Guntas Korkmaz et al. [6] investigated TAS and markers of oxidative stress in subjects with normal or impaired glucose regulation in patients with diabetes. They found that hyperglycemia was related to increased ischemia-modified albumin, advanced oxidation protein products, and prooxidants-antioxidants balance concentrations, and an increase in glucose concentrations during glucose loading could cause tissue damage by increasing oxidative stress. Serin et al. [7] showed that postchallenge 2 h serum thiobarbituric acid reactive substances and oxidized low density lipoprotein levels in subjects with impaired glucose tolerance and in the diabetic glucose tolerance groups were found to be higher than their baseline levels, which might suggest that oxidative stress occurs at an early stage in diabetes. In this study, oxidative stress markers increased significantly in all the groups and antioxidant markers decreased significantly. The difference between the results of the studies could be explained by using different antioxidative and oxidative stress markers in determining the oxidative stress status in the body.

Choi et al. [8] investigated 16 plasma markers as indicators of inflammation and oxidative stress during the OGTT in 54 individuals. Leptin, retinol-binding protein-4, CRP, osteopontin, angiogenin, macrophage-derived chemokine, and macrophage colony stimulating factor concentrations significantly decreased during the OGTT, while IL-6, IL-8, and monocyte chemoattractant protein-3 concentrations significantly increased during the OGTT, which might support that glucose ingestion has an impact on systemic inflammation and oxidative stress. Manning et al. [9] measured the levels of inflammatory cytokines (IL-6, TNF-α) and peroxides as oxidative stress markers during the OGTT performed for 33 overweight or obese individuals at 0., 30., 60., 90., and 120. min. They found that IL-6 decreased at 30. and 60. min, while peroxides decreased significantly at 60. min as compared to the baseline. Contrary to the results of this study, we found that oxidative stress markers (TOS, LOOH) significantly increased.

Andreeva-Gateva et al. [10] investigated SOD, glutathione, and TAS levels as antioxidant parameters in 36 healthy volunteers and 36 patients with metabolic syndrome at 0., 60. and 120. min of the OGTT. They found significant decreases in SOD and glutathione peroxidase activity at 120. min in both groups while the TAS was increased significantly at 120. min. In this study, the TAS value was significantly lower at both 60. and 120. min. 

Ceriello et al. [11] demonstrated that antioxidant defenses were reduced during the OGTT both in normal and noninsulin-dependent diabetic subjects. Similarly, we also found that the OGTT resulted in a significant decrease in antioxidant defense in both diabetic and nondiabetic patients in this study. Nakanishi et al. [12] examined serum glucose and urine isoprostane as oxidative stress markers at the baseline, the 1st hour, and the 2nd hour of the 75 g OGTT in 775 Japanese-American individuals who had normal glucose tolerance, impaired glucose tolerance, or diabetes. They found glucose excursion might lead to oxidative stress, which is similar to the results of this study. 

Muratoğlu et al. evaluated the effect of the 50 g glucose challenge test (GCT) on thiol/disulfide balance in 100 women at 24–28 weeks of gestation [13]. The glucose load increased oxidative stress by changing the thiol/disulfide homeostasis in GCT-positive pregnant individuals while not in healthy pregnancies. Gelaleti et al. demonstrated that increased oxidative DNA damage was present in patients with overt gestational diabetes and mild gestational hyperglycemia [14]. On the other hand, Rueangdetnarong et al. [15] compared the levels of oxidative stress biomarkers between pregnancies with gestational diabetes mellitus (GDM) or without GDM. They reported that pregnancies with GDM had increased oxidative stress and apoptosis markers levels, which were not correlated with pregnancy outcomes. Better glycemic control did not prevent the increase in oxidative stress. Biomarker levels in cord blood of pregnant women with GDM were not altered, which suggested that the placenta could be the barrier for the oxidative stress and cytokines.

Although there are many studies carried out on many patient groups using many biomarkers to evaluate the relationship between the OGTT and oxidative stress and their effects on the human body, diverse results have been reported, some of which are contrary to each other. In our opinion, such different results may be mainly attributed to the use of many diverse antioxidative and oxidative stress markers to determine the oxidative stress status of the body. To avoid this, reaching a consensus on which antioxidative and oxidative stress markers should be used to determine the oxidative stress status of the body, and measuring these determined markers, will be a good approach.

We aimed to address the issue of whether to perform the OGTT in pregnant women for screening purposes. When planning the study, we were hoping to find out that OGTT screening would not result in any significant increase in oxidative stress in humans and conclude that the OGTT could also be safely carried out in pregnant women. However, we found that the OGTT increased oxidative parameters and decreased antioxidative parameters in humans, consequently causing an increase in oxidative stress. However, since we did not evaluate whether these biomarkers are present in cord blood of pregnant women, we do not know their effects on fetuses. This will require further studies regarding the effects of the OGTT on humans and particularly on pregnant women, whether the oxidative stress in the pregnant women affects fetuses or not, and, if it does, what the effects will be. Furthermore, studies are also required to determine whether the hyperglycemia peak caused by the OGTT in pregnant women will result in a hyperglycemia peak in the fetus, and if it does, whether oxidative stress and possible related damage will result.
